# Effects of Subminimum Inhibitory Concentrations of Antibiotics on the *Pasteurella multocida* Proteome: A Systems Approach

**DOI:** 10.1155/2008/254836

**Published:** 2008-04-22

**Authors:** Bindu Nanduri, Mark L. Lawrence, Divya Swetha Peddinti, Shane C. Burgess

**Affiliations:** ^1^College of Veterinary Medicine, Mississippi State University, Mississippi State, MS 39762, USA; ^2^Mississippi State University Institute for Digital Biology, Mississippi State University, Mississippi State, MS 39762, USA; ^3^Mississippi Agriculture and Forestry Experiment Station, Mississippi State University, Mississippi State, MS 39762, USA; ^4^MSU Life Sciences and Biotechnology Institute, Mississippi State University, Mississippi State, MS 39762, USA

## Abstract

To identify key regulators of subminimum inhibitory concentration (sub-MIC) antibiotic response in the *Pasteurella multocida* proteome, we applied systems approaches. Using 2D-LC-ESI-MS^2^, we achieved 53% proteome coverage. To study the differential protein expression in response to sub-MIC antibiotics in the context of protein interaction networks, we inferred *P. multocida* Pm70 protein interaction network from orthologous proteins. We then overlaid the differential protein expression data onto the *P. multocida* protein interaction network to study the bacterial response. We identified proteins that could enhance antimicrobial activity. Overall compensatory response to antibiotics was characterized by altered expression of proteins involved in purine metabolism, stress response, and cell envelope permeability.

## 1. Introduction

The use of antibiotics is continually being challenged by the emergence of resistant strains of bacteria [1]. Resistance has resulted both from mutations associated with bacterial DNA replication and from horizontal gene transfer [2, 3]. However, while antibiotic resistance is increasing, progress in the discovery of new antimicrobial compounds is not [4]. The current trends in drug resistance and drug discovery could result in future crises in treating microbial infections.

Currently, antibiotic therapy is based on achieving and exceeding a minimum inhibitory concentration (MIC) for a sufficient amount of time in infected tissues [5]. The ability to surpass and exceed greater than MIC in target tissues is influenced by the susceptibility of the pathogen to a given antibiotic and the ability of the antibiotic to partition into the target tissue. However, when faced with an infection caused by an antibiotic-resistant bacterial strain, it may not be possible to surpass the MIC in the target tissue. In this situation, one therapeutic option is treatment with a sub-MIC concentration of the antibiotic. Sub-MIC antibiotic therapies can, however, lead to treatment failure and antibiotic resistance [2]. Therefore, careful evaluation of sub-MIC effects on bacterial physiology is needed prior to therapeutic use of sub-MICs.

*Pasteurella multocida* is an important Gram-negative zoonotic respiratory bacterial pathogen with a broad host range [6–10], and it is a particularly good model organism to study antibiotic effects because it has a Gram-negative envelope that is permeable to hydrophobic molecules (including antibiotics) [11, 12]. Therefore, investigating antibiotic effects is possible with fewer confounding effects caused by permeability differences between antibiotics.

Sub-MICs of antibiotics model the conditions that bacteria face in wild environments and have evolved to survive [13]. Sub-MICs of antibiotics cause defined effects on, and responses in, bacterial physiology. For example, although sub-MICs of chlortetracycline reduced virulence factor expression in the bovine respiratory pathogen *Mannheimia haemolytica* [14] they caused secondary or “nontarget” effects, in addition to their primary target effects, in *Pasteurella multocida* [15]. Some of these secondary effects may enhance the primary activity of the antibiotic, while others, such as an increased expression of recombinase A (RecA) in response to enrofloxacin, are apparent compensatory mechanisms.

Molecular systems level analysis including interaction networks can identify not only direct and indirect global responses of bacterial genes to sub-MICs, but may also identify key elements in genetic networks (regulatory hubs) that, when altered, change the fundamental properties of these networks [16]. Identification of key targets in the context of sub-MIC antibiotic effects on virulence factors necessary for pathogenesis, and leading to antimicrobial resistance, can help assess the benefit versus risk of sub-MIC therapeutic usage. Systems approaches can also identify key molecules as potential targets for novel therapeutic combinations.

Here, we report a systems analysis of the *Pasteurella multocida* Pm70 response to sub-MICs of three different antibiotics that differ in their mode of action: amoxicillin (AMX), which is a cell wall biosynthesis inhibitor; chlortetracycline (CTC), which inhibits protein synthesis; enrofloxacin (ENR), which is a quinolone that inhibits DNA gyrase and DNA topoisomerase IV [17]. We used three different classes of antibiotics to find common themes to antibiotic effects and the bacterial adaptive response. Of these three antibiotics, resistant strains of *P. multocida* are described for penicillins and tetracyclines [18]. Currently, no fluoroquinolone resistant *P. multocida* are described; however, resistance to this antibiotic is documented for other Gram-negative pathogens [19]. Using a liquid chromatography tandem mass spectrometric proteomics approach, we identified 53% of the predicted *P. multocida* proteome expressed under our experimental conditions. The protein expression data was analyzed in the context of interaction networks using Pathway Studio (Ariadne, Rockville, Md, USA), which has canonical bacterial interaction networks. Overall compensatory response of *P. multocida* to sub-MICs was characterized by altered expression of virulence factors; proteins involved in purine metabolism, stress response, and cell envelope permeability.

## 2. Materials and Methods

### 2.1. Pasteurella Multocida Culture

*Pasteurella multocida* strain Pm70, a serotype A : 1 poultry isolate that has a fully sequenced genome [20], was used in this study. Pm70 was cultivated in brain heart infusion (BHI) broth at 37°C with rotary aeration. Minimum inhibitory concentrations (MICs) of AMX, CTC, and ENR for Pm70 are 0.5 *μ*g/mL, 4 *μ*g/mL, and 0.031 *μ*g/mL, respectively [15]. Growth kinetics of Pm70 in the presence of 1/4 MIC of the three antibiotics were previously described [15]. Stationary phase cultures of Pm70 were used to inoculate 50 mL BHI to an initial A_600_ of 0.05; antibiotic treated cultures contained 1/4 MIC of AMX, CTC, or ENR, and control cultures were grown without antibiotics. All cultures were grown in triplicate to mid-log phase (A_600_ of 0.8) and harvested by centrifugation (10 000 × g, 10 minutes, 4°C). Pellets were stored at −80°C.

### 2.2. Proteomics

Protein extraction, quantification, and trypsin digestion were done exactly as described [14] from three biological replicates. Briefly, protein solutions (100 *μ*g; <1 M urea; 50 mM Tris-cl pH 8.0) from untreated control and antibiotic-treated bacteria were reduced (5 mM dithiothreitol, 65°C, 5 minutes), alkylated (10 mM iodoacetamide, 30°C, 30 minutes), and then trypsin-digested until there was no visible pellet (1 : 50 w/w 37°C, 16 hours). Peptides were desalted using a peptide macrotrap (Michrom BioResources, Inc., Auburn, Calif, USA) and eluted using a 0.1% triflouroacetic acid, 95% ACN solution. Desalted peptides were vacuum dried and resuspended in 20 *μ*l of 0.1% formic acid.

Two-dimensional liquid chromatography (LC) analysis was done by strong cation exchange (SCX) followed by reverse phase (RP) coupled directly in line with an electrospray ionization (ESI) ion trap tandem mass spectrometer (LCQ; ThermoElectron Corp., San Jose, Calif, USA) essentially as described in [15]. The salt gradient applied in this study was different from the published method and was applied in steps of 0, 10, 15, 20, 25, 30, 35, 40, 45, 50, 57, 64, 90, and 700 mM ammonium acetate in 5% acetonitrile (ACN) and 0.1% formic acid. The reverse phase gradient used 0.1% formic acid in ACN and increased the ACN concentration in a linear gradient from 5% to 30% in 20 minutes and then 30% to 95% in 7 minutes, followed by 5% for 10 minutes for 0, 10, 15, 25, 30, 45, 64, 90, and 700 mM salt gradient steps. For 20, 35, 40, 50, and 57 mM salt gradient steps, ACN concentration was increased in a linear gradient from 5% to 40% in 65 minutes, 95% for 15 minutes, and 5% for 20 minutes.

All database searches of tandem mass spectra were done using TurboSEQUEST (Bioworks Browser 3.2; ThermoElectron) [21]. Mass spectra and tandem mass spectra were searched against an in silico trypsin-digested protein database of *P. multocida* Pm70 downloaded from National Center for Biotechnology Institute (NCBI). Cysteine carbamidomethylation and methionine oxidation (single and double) were included in the search criteria. We used the reverse database functionality in Bioworks 3.2 and searched tandem MS (MS^2^) data against a reversed Pm70 database using the same search criteria as described above. Peptide identifications from Pm70 protein database that were >5 amino acids long with Xcorr ≥ 1.5, 2.0, and 2.5 for +1, +2, and +3 charged ions, respectively, and with delta Cn values of ≥0.1 were used for protein identifications [15]. None of the proteins identified at the applied Xcorr and delta Cn filters had peptides identified from the reversed Pm70 database. Protein identifications have been submitted to PRoteomics IDEntifications (PRIDE) database [22] and the accession numbers are 1751, 1752, 1753, and 1754. PRIDE submission requirements are based on the proposed guidelines by proteomics standards initiative [23] and include all the peptides identified for each protein with their sequence, charge state, Xcorr, and delta cn.

We used an isotope-free quantification method [14] and a custom program *ProtQuant* [24] to identify differences in protein expression between control and sub-MIC antibiotic treated *P. multocida. ProtQuant* is a java-based tool for label-free quantification that uses a spectral counting method [25] with increased specificity. *ProtQuant* includes the quantitative aspects of the Sequest cross correlation (XCorr) into the spectral counting method and computes the statistical significance of differential protein expression using one-way ANOVA (*α* ≤ 0.05). This method requires at least 3 peptides identified from either the control or treatment datasets.

### 2.3. Systems Modeling: Pasteurella Multocida Pm70 Molecular Interaction Database

To study the effects of antibiotics on Pm70 using systems biology approaches, we built an information rich predicted protein interaction network using Pathway Studio (Ariadne, Rockville, Md, USA) using a bacterial molecular interaction database. Multiple aspects of protein function, including protein modifications, cellular location, protein-protein interactions, gene expression regulation, molecular transport and synthesis, and regulation of various cellular processes are included [26].

Although the bacterial molecular interaction database contains data for Gram-negative and Gram-positive bacteria, it does not include *P. multocida*. Therefore, to append the bacteria database with *P. multocida*, all 2015 Pm70 proteins were mapped to their corresponding orthologs in the database by identifying reciprocal-best-BLAST hits with greater than 30% similarity. Gram-positive orthologs were removed to ensure that protein interaction networks were derived only from Gram-negative species. The resulting ortholog map file was imported into Pathway Studio to allow prediction of interactions between *P. multocida* proteins. The Gram-negative ortholog-only network was used to analyze sub-MIC antibiotic effects on the *P. multocida* proteome. Our protein quantification method did not measure absolute fold changes in protein expression, but instead indicated whether the increase or decease in expression was significant. Therefore, for each antibiotic treatment, we imported all identified proteins into Pathway Studio along with the expression values indicating the significance of expression change; we represented a significant increase in protein expression compared to the untreated control as +1, while a significant decrease in expression was represented as −1.

As an initial screening method, we used the Pathway Studio “Find groups” tool to identify Gene Ontology (GO) groups that had a significant number of identified proteins within each paired antibiotic treatment/control protein set. By doing so we identified GO groups that had good proteome coverage for analysis of antibiotic effects. Pathway Studio calculates the statistical significance of the overlap between the protein list and a GO group using the Fisher exact test. Thus, the calculated p-value depends on the extent of overlap between the protein list and a group as well as the sizes of the list and a group. We used *P* ≤ .05 to select GO groups with significant protein coverage.

We built interaction networks in Pathway Studio with proteins of interest including the upstream regulators and downstream targets. In the interaction networks, different colors were used for the nodes to indicate whether a protein was present in the dataset (pink) and whether there was a significant increase (red), or decrease (green) in protein expression in response to sub-MIC of antibiotic. Entities in the interaction map that were not from Pm70 were shown in gray color. For each of the three antibiotics, protein expression was compared between the control (no antibiotic) and the antibiotic treatment and overlaid onto the Pm70 interaction network. The individual responses to each antibiotic were compared to identify overall trends in Pm70 response to the different antibiotics.

## 3. Results and Discussion

### 3.1. Proteome Coverage

We identified 1064 (53%) of the 2015 predicted Pm70 proteins [20] from our control (no antibiotic) and antibiotic treated datasets. The number of identifications with two or more peptides in at least one dataset was 572 (56%) (PRIDE datasets (supplementary Table 1)) and this compares favorably with reported MudPIT results which have as few as 20% of proteins identified by two or more peptides [27]. Furthermore, compared to the 20% coverage of the accessible proteome that we reported with ICAT [15], we achieved 2.5 fold higher proteome coverage in this study. This level of proteome coverage gave us confidence that we could conduct a systems-wide analysis to formulate new hypothesis for sub-MIC antibiotic effects on Pm70 physiology.

**Figure 1:**
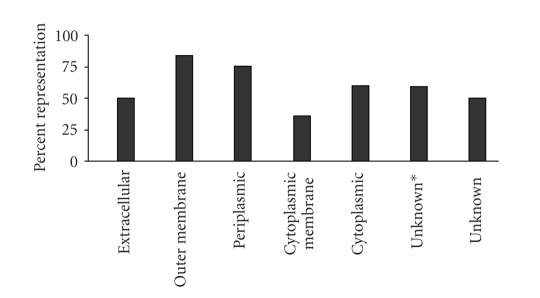
Representation of *P. multocida* proteins in PSORTb subcellular locations. Percent coverage for each PSORTb predicted subcellular localization for *P.multocida* proteins identified in this study is shown. Percent representation was calculated by comparing the number of proteins identified in each category to the total number of proteins predicted to be in that category from *P. multocida* genome. PSORTb prediction “unknown*” indicates that the proteins may have multiple subcellular localizations while unknown refers to location unknown.

Furthermore, because protein function depends on cellular location, a systems-wide analysis requires that we identify proteins from all cell locations. We used the PSORTb version 2.0 bacterial protein subcellular localization prediction tool to classify all proteins in our dataset [28]. PSORTb has an overall precision of 96% and places all Pm70 proteins into seven categories: extracellular (4), outer membrane (49), periplasmic (61), cytoplasmic membrane (411), cytoplasmic (774), unknown (with multiple localization sites) (37), and unknown (679). To determine the percent coverage in each of these categories, the number of proteins identified in each category was compared to the total number of proteins predicted to be in that category from the genome (Figure 1). The average coverage for each subcellular compartment in our data was 59%, and although proteins from all subcellular compartments were represented, not all subcellular compartments were equally represented. We identified more outer membrane and periplasmic proteins (84% and 75%, resp.), than cytoplasmic membrane proteins (36%).

To investigate the lower coverage of cytoplasmic membranes, we estimated the total abundance of cytoplasmic membrane and outer membrane proteins based on ΣXcorr [14]. The outer membrane proteins had a ΣXcorr of 1599, while cytoplasmic membrane proteins had a ΣXcorr of 1424. Therefore, although a lower percentage of cytoplasmic membrane proteins were identified, there was a very similar amount of protein isolated from the cytoplasmic and outer membrane fractions. This suggests that although the Pm70 cytoplasmic membrane contains a greater variety of proteins than the outer membrane, cytoplasmic membrane proteins are present in relatively low abundance compared to the outer membrane. Because we used the mass spectrometer in a data-dependent way to maximize proteome coverage, it is logical that we achieved greater coverage of bacterial compartments that have proteins with higher abundance. These results also show that our methods for protein isolation solubilized relatively hydrophobic membrane proteins. Our previously published ICAT methodology [15] and the current label-free 2D-LC ESI MS^2^ approach utilized the same protein isolation method. Nonisotopic relative quantitative proteomics methods are as good (or better) than isotopic methods [14, 29] but have the advantage of far greater proteome coverage. We confirm this observation in the current work; compared to our previous ICAT study we identified tenfold more membrane proteins, which is 38.6% of the predicted membrane proteome of *P. multocida* compared with 3.7% identified using the ICAT method.

### 3.2. Pasteurella Multocida Pm70 Molecular Interaction Database

Predicted protein interaction networks for *P. multocida* (based on computational comparative genomics) are available [30]. However, these interactions have edges that have no directionality or any biological information. System analysis can be a powerful tool to identify global trends so long as the protein interaction network under investigation is information rich. Therefore, to enable system analysis of our protein expression data, we chose to build a protein interaction network for *P. multocida* where all nodes had all possible biological information associated with them. Reciprocal-BLASTp searches of Pm70 proteins against both Gram-negative and Gram-positive bacterial proteins identified unique orthologs for only 1595 proteins. Of these, 1525 were from Gram-negative bacteria (*Escherichia coli* CFT 073, *E. coli* K12, *E. coli* O157:H7, *Synechocystis* sp. PCC6803 (mean *e*-value and percent identity were 2.03e-06 and 65.2%) of which 848 orthologs were identified in our datasets. We constructed a network with 848 proteins in which the nodes (proteins) had predicted functional annotation and the edges (links) between proteins described regulation, expression, binding, and chemical reactions.

### 3.3. Systems Modeling: Data Analysis

To determine the effects of 1/4 MIC of each of the three antibiotics, AMX, CTC, and ENR, on the Pm70 proteome, protein expression from each of the three antibiotic treatments was compared to protein expression from the nonantibiotic control. We did not compare protein expression changes between antibiotic treatments. The paired control antibiotic-treated samples contained 913, 781, and 762 proteins, representing 143, 133, and 137 GO groups, from AMX-, CTC-, and ENR-treated cultures, respectively. Each GO group is a unique GO term that corresponds to a biological process, molecular function, or a cellular component annotation.

At *P* ≤ .05, 69, 61, and 58 GO groups for AMX, CTC, and ENR, respectively had significant proteome coverage. Of these, 46 GO groups were common to all three antibiotics (Table 1). To identify the underlying higher level themes involved in the response to antibiotics, we used the *GoSlimViewer* at AgBase [31] and found that the 46 common GO groups had 16 higher level GO terms (Table 2).

We identified 147, 126, and 134 significant changes in protein expression with sub-MIC AMX, CTC, and ENR, respectively (Table 3, and supplementary Table 2, all proteins were identified with at least three peptides in one dataset). A number of proteins differentially expressed in response to sub-MIC antibiotics in our previous ICAT study were also identified in this study. Although the general trend of expression was similar (i.e., increase or decrease) compared to ICAT, many of the differences were not statistically significant in this study. This could be due to the data dependent acquisition inherent to ESI MS^2^ methodology combined with the differences in both the amount of the proteome accessible by ICAT and label-free proteomics and the difference in total coverage (numbers of peptides/protein). We suggest that this nonisotopic proteomics workflow and analysis offer more comprehensive coverage and a greater probability of accurate representation of the Pm70 proteome than did our ICAT study.

To identify common themes in the response to sub-MICs of antibiotics, we superimposed significant changes in protein expression for each antibiotic onto the Pm70 protein interaction network (supplementary Figure ). For more detailed analysis of selected proteins, we used the Pm70 protein interaction network to iteratively build and visualize networks around the proteins of interest. The network built around RecA in response to ENR is shown in Figure 3 as an example of the type of analysis that was used to identify the trends described in the following paragraphs.

### 3.4. Common Adaptive Responses to AMX, CTC, and ENR

**Figure 2:**
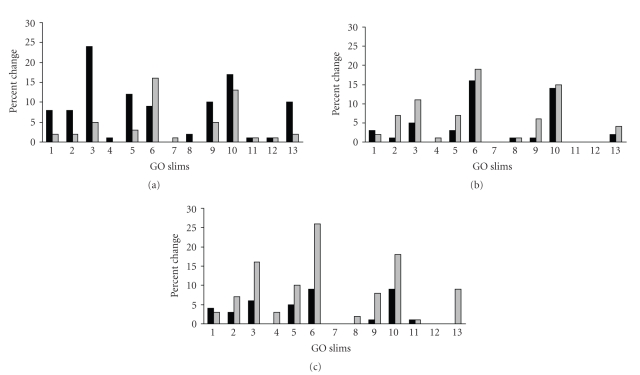
Overall trends in protein expression in GO Slims common to sub-MICs. For each GO Slim, the percentage of proteins whose expression was either up (black bars) or down regulated (gray bars) is compared to the total entities in that GO Slim are shown for amoxicillin (a), chlortetracycline (b), and enrofloxacin (c). GO Slim categories are as follows: 1, external encapsulating structure; 2, DNA metabolism; 3, nucleobase, nucleoside, nucleotide, and nucleic acid metabolism; 4, protein metabolism; 5, transcription; 6, protein biosynthesis; 7, response to stress; 8, carbohydrate metabolism; 9, catabolism; 10, structure molecule activity; 11, peptidase activity; 12, amino acid derivative metabolism; 13, generation of precursor metabolites and energy.

**Figure 3:**
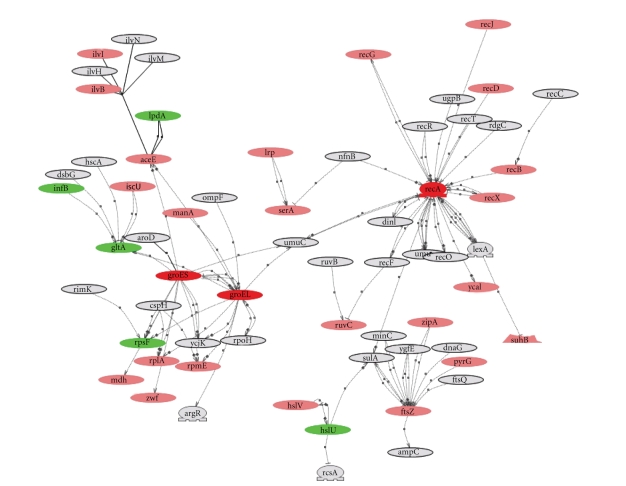
*P. multocida* RecA protein interaction network. *P. multocida* sub-MIC ENR response was marked by significant change in RecA expression. We built interaction network iteratively with RecA as primer and identified RecA, GroEL, and GroES subnetwork. Red nodes are proteins with increased expression and green nodes are proteins with decreased expression in response to ENR. Proteins with no significant changes in expression are shown in pink, and gray nodes are proteins from *P. multocida* interaction network that were not identified in our dataset.

We examined the overall trends of antibiotic effects on the 16 GO groups that were identified by *GO SlimViewer* (Table 2). After excluding general GO terms like cytoplasm, metabolism and so forth, 13 GO groups were evaluated. For each GO group, we calculated the percentage of proteins whose expression either increased or decreased, compared to the total entities in that GO group, for each antibiotic treatment. The trend of AMX was an overall increase in protein expression, which could indicate the induction of an adaptive response (Figure 2). Conversely, CTC and ENR had an overall suppressive effect on protein expression, potentially indicating that CTC and ENR detrimentally affect *P. multocida* fitness at doses below MIC. Alternatively, this could be a compensatory response by slowing metabolism.

The overall suppressive effects of CTC and ENR on *P. multocida* metabolism are further substantiated by their effects on individual protein expression in central metabolic pathways. In glycolysis/gluconeogenesis, CTC and ENR significantly decreased the expression of phosphoenolpyruvate carboxykinase, phosphoglycerate kinase, fructose bisphosphate aldolase, and glyceraldehyde-3-phosphate dehydrogenase. By contrast, AMX significantly increased the expression of fructose bisphosphate aldolase, pyruvate kinase, and phosphoglyceromutase, and it significantly increased expression of fumarate hydratase and succinyl-CoA synthetase from the TCA cycle (Table 3).

The differential protein expression profiles of *P. multocida* in response to antibiotic sub-MICs were analyzed to delineate the underlying physiological response. A predictable stress response was indicated by increased GroES expression after treatment with all three antibiotics and increased GroEL and DnaK expression after AMX treatment. The GroEL/GroES chaperone system is induced by different forms of environmental stress in various bacterial species and functions to maintain appropriate protein folding [32]. All antibiotics reduced the expression of FtsH, a zinc metalloprotease that degrades cellular proteins, including heat shock promoter protein sigma 32 (RpoH) [33]. Therefore, sub-MICs of antibiotics may reduce expression of *P. multocida* genes under the control of heat shock promoters.

Sub-MICs of antibiotics induce a response that appears to decrease cell envelope permeability by increasing the availability of N-acetyl glucosamine, a necessary precursor for peptidoglycan and lipid A biosynthesis. *E. coli* mutants that are defective in lipid A biosynthesis are permeable to hydrophobic molecules and highly susceptible to antibiotics [34, 35]; therefore, increasing the amount of lipid A would be a logical response to decrease permeability. All three antibiotics increased GlmS expression, which catalyzes the first reaction in the pathway for N-acetylglucosamine biosynthesis, and AMX increased GlmU expression, which catalyzes the last two reactions in the pathway. NagC, is a transcriptional regulator controlling *glmUS* operon expression [36], its expression decreased after CTC and ENR treatment. In addition to increasing N-acetylglucosamine availability, CTC and ENR decreased expression of OmpA, a multifunctional outer membrane porin that allows diffusion of small solutes across the outer membrane [37, 38] (Table 3).

Purine metabolism was another common cellular function affected by all three antibiotics. AMX, CTC, and ENR increased adenylate kinase expression. CTC and ENR increased adenylosuccinate synthetase expression. Adenylate kinase and adenylosuccinate synthetase catalyze the first and last steps in the conversion of inosine monophosphate to adenosine diphosphate [39], respectively. AMX and CTC also significantly increased expression of PrsA, which catalyzes formation of phosphoribosyl pyrophosphate, a necessary metabolite for synthesis of purines, pyrimidines, and histidine.

RNA metabolism was affected by antibiotics; all increased RNA polymerase expression; AMX increased RpoA, RpoB, RpoC, and RpoZ expression; CTC increased RpoA expression; and ENR increased RpoZ expression. In addition, all three antibiotics decreased expression of transcriptional terminator Rho, which may cause polarity suppression. Decreased expression of Rho may also decrease the half life of mRNA in the bacterial cell [40].

It is possible that these protein expression trends in *P. multocida* that were common for all three antibiotics could reflect a general response to stress. These changes could be induced by other nonantibiotic stressors; further experiments with nonantibiotic stress conditions would be required to confirm that these trends are in fact a common response to antibiotic-induced stress.

### 3.5. Virulence Factor Expression

Sub-MICs of antibiotics can potentially impact the outcome of infection by altering bacterial virulence factor expression [14, 41–43]. In the current study, antibiotics altered expression of known and putative virulence proteins in *Pasteurella multocida*. For example, CTC and ENR reduced the expression of OmpA, which in addition to allowing small molecule diffusion across the outer membrane, is also a known virulence factor of *P. multocida* and is involved in binding to host cells [44]. In addition, sub-MICs of CTC and ENR decreased expression of detoxifying enzymes superoxide dismutase and catalase, which constitute a major defense mechanism of bacteria against reactive oxygen species and play a role in pathogenesis in certain bacteria [45, 46]. Other putative *P. multocida* virulence factors whose expression was decreased by CTC and ENR included glyceraldehyde-3-phosphate dehydrogenase, which is a virulence factor in Gram-positive bacteria and has been recently implicated in pathogenesis of Gram-negative bacteria [47], and phosphoenolpyruvate carboxykinase, which is required for *M. bovis* virulence in mice [48]. Stress response chaperone protein Hsp90 was decreased by CTC and ENR, and DnaK was decreased by ENR. These proteins are required for virulence in a number of bacterial pathogens including *S. enterica* and *L. pneumophila* [49, 50].

### 3.6. Amoxicillin Specific Effects

The AMX mode of action involves binding and inactivation of peptidoglycan cross-linking transpeptidases with subsequent inhibition of cell wall biosynthesis. The transpeptidases, also known as penicillin binding proteins (Pbps), are known targets of AMX [51] and the *P. multocida* genome has eight Pbps [20]. Logically, the bacterial compensatory response to AMX could involve increasing Pbp expression. Though we identified 5 Pbps in our dataset; there were no significant changes in expression of any of these. Therefore, it appears that the adaptive response to AMX treatment does not entail over expression of Pbps. Alternatively, more than 1/4 MIC of AMX may be required to elicit over expression of Pbps in *P. multocida*.

There was good evidence that the *P. multocida* compensatory response to AMX involves decreasing cell envelope permeability. In addition to the previously described effects on GlmU and GlmS, expression of several enzymes responsible for synthesis of lipopolysaccharide was significantly increased. LpxB, an enzyme in the lipid A biosynthetic pathway, had significantly increased expression. KdsA and RfaE, both of which are involved in synthesis of core oligosaccharide [52], were significantly increased. Expression of GalU, which is responsible for the synthesis of cell envelope precursor UDP-galactose, was also increased.

The compensatory response to AMX also appeared to involve upregulation of cell division proteins. Both FtsZ (essential for cell division) and FtsY had increased expression. AMX also caused increased expression of DeoR: a DNA-binding transcriptional regulator involved in the negative regulation of genes encoding nucleotide catabolism enzymes.

### 3.7. Chlortetracycline Specific Effects

As could be predicted, the response to CTC involved the compensation against its inhibition of protein translation. Elongation factor Tu was increased, as was methionyl-tRNA synthetase (MetG), which initiates translation through tRNA(fMet) aminoacylation. In addition, DeaD (CsdA), which participates in the assembly of the large subunit of the ribosome [53], also had increased expression.

CTC also appeared to induce a protective response for the cell envelope. In addition to its effects on GlmS, NagC, and OmpA, it caused an increase in expression of murein lipoprotein Lpp, which is required for stabilization and integrity of the cell envelope [54]. Lpp mutants are hypersensitive to toxic compounds [55]. Finally, CTC caused increased expression of Lrp, a transcriptional regulator that is a global mediator of the leucine response, specifically branched amino acid transport [56].

### 3.8. Enrofloxacin Specific Effects

Results from the current study agree with our earlier findings [15] that the compensatory response to ENR entails recruitment of double stranded DNA damage repair machinery to overcome the quinolone-mediated block on progression of the replication fork. This response is logical because blockage of the replication fork by quinolones is caused by accumulation of inactive quinolone-bound intermediate with double stranded DNA breaks. We previously reported an increase in expression of RecA, which catalyzes DNA strand exchange during homologous recombination and double-stranded DNA break repair [15], in response to ENR. Here we found that expression of RecA and RecN, another enzyme required for repair of double stranded breaks in the chromosome, increased.

## 4. Conclusions

We achieved greatly improved coverage of the *P. multocida* proteome by detecting unlabeled peptides using 2D LC with ESI MS^2^ compared to a similar study we conducted using isotope coded affinity tag labeled (ICAT) peptides [15]. The advantage of ICAT labeling is that we were able to report fold changes in protein expression; however, the increased proteome coverage in the current study enabled us to conduct systems analysis, which was not possible in the ICAT study.

Systems analysis improved our study of *P. multocida* protein expression and enabled the identification of antibiotic-mediated mechanisms and pathways. In particular, visualizing proteins as interacting networks enabled us to identify proteins with high connectivity that could be important targets for modulation. However, although proteome coverage was not a major limitation in conducting systems analysis with our *P. multocida* protein expression data, the paucity of experimental evidence from this nonmodel species did limit our analysis. The strategy we used to identify orthologs and import experimental evidence based on *E. coli* and other model species greatly enhanced our analysis, but extrapolation of protein functions across species must be interpreted in the light of this electronic inference.

Despite this limitation, our systems analysis identified several proteins to target in future studies. In particular, we speculate that inactivating *P. multocida* proteins in the heat shock response, cell envelope biosynthesis/integrity, purine metabolism, or RNA metabolism may be viable strategies to enhance antimicrobial activity. In addition, inactivation of specific cell division proteins, translation proteins, and DNA repair enzymes may impair bacterial adaptation to antibiotics in the penicillin, tetracycline, and quinolone classes, respectively. Inactivation of specific transcriptional regulators, such as DeoR or Lrp, may also be an effective method to impair the *P. multocida* adaptive response against AMX and CTC. These targets would not have been predicted a priori based on the known mechanisms of action of these antibiotics; their identification was facilitated by our global systems analysis.

In summary, our findings could lead to the development of new antimicrobial potentiating drugs aimed at inactivating antibiotic compensatory mechanisms to prevent occurrence and emergence of antimicrobial resistance. In addition, our results indicate that systems analysis in nonmodel bacterial species can be used with high-throughput proteomics data to identify protein targets for further functional investigations.

## Supplementary Material

All proteins identified in this study are listed in Supplementary Table 1. Proteins with significant quantitative changes in expression in response to sub-MICs of AMX, CTC, or ENR are listed in Supplementary Table 2. Protein interaction networks built with significant changes in expression with sub-MICs shown in Table 3 are included in the supplementary figure. This figure shows changes in protein expression in response to sub-MICs of AMX, CTC, and ERN. Red and green indicate significant increase and decrease in expression, respectively. Proteins whose expression did not change (pink) and proteins in the network not identified in our datasets (gray) are also included.

## Figures and Tables

**Table 1:** Significantly overrepresented GO groups in response to sub-MIC AMX, CTC and ENR.

GO ID	Name
GO:0009257	10-formyltetrahydrofolate biosynthesis
GO:0045733	Acetate catabolism
GO:0006086	Acetyl-CoA biosynthesis from pyruvate
GO:0006418	Amino acid activation
GO:0009063	Amino acid catabolism
GO:0046349	Amino sugar biosynthesis
GO:0009061	Anaerobic respiration
GO:0015986	ATP synthesis coupled proton transport
GO:0030113	Capsule (sensu Bacteria)
GO:0016052	Carbohydrate catabolism
GO:0042280	Cell surface antigen activity, host-interacting
GO:0017004	Cytochrome biogenesis
GO:0005737	Cytoplasm
GO:0009281	Cytosolic ribosome (sensu Bacteria)
GO:0006308	DNA catabolism
GO:0006261	DNA dependent DNA replication
GO:0006310	DNA recombination
GO:0006113	Fermentation
GO:0006012	Galactose metabolism
GO:0006094	Gluconeogenesis
GO:0006096	Glycolysis
GO:0009436	Glyoxylate catabolism
GO:0009089	Lysine biosynthesis via diaminopimelate
GO:0009086	Methionine biosynthesis
GO:0006777	Mo-molybdopterin cofactor biosynthesis
GO:0015949	Nucleobase, nucleoside and nucleotide interconversion
GO:0009052	Pentose-phosphate shunt, non-oxidative branch
GO:0009051	Pentose-phosphate shunt, oxidative branch
GO:0008233	Peptidase activity
GO:0009252	Peptidoglycan biosynthesis
GO:0000270	Peptidoglycan metabolism
GO:0042597	Periplasmic space
GO:0006412	Protein biosynthesis
GO:0006457	Protein folding
GO:0006508	Proteolysis and peptidolysis
GO:0006164	Purine nucleotide biosynthesis
GO:0009152	Purine ribonucleotide biosynthesis
GO:0042867	Pyruvate catabolism
GO:0009269	Response to dessication
GO:0006401	RNA catabolism
GO:0009451	RNA modification
GO:0003735	Structural constituent of ribosome
GO:0009088	Threonine biosynthesis
GO:0006350	Transcription
GO:0009386	Translational attenuation
GO:0006099	Tricarboxylic acid cycle

AMX: amoxicillin; CTC: chlortetracycline; ENR: enrofloxacin.

**Table 2:** GO slims common to sub-MIC AMX, CTC, and ENR response of *P. multocida*.

GO ID	GO TERM
*Cellular Component *	
GO:0005737	Cytoplasm
GO:0005829	Cytosol
GO:0030312	External encapsulating structure

*Biological Process *	
GO:0006519	Amino acid and derivative metabolism
GO:0005975	Carbohydrate metabolism
GO:0009056	Catabolism
GO:0006259	DNA metabolism
GO:0006091	Generation of precursor metabolites and energy
GO:0008152	Metabolism
GO:0006139	Nucleobase, nucleoside, nucleotide and nucleic acid metabolism
GO:0006412	Protein biosynthesis
GO:0019538	Protein metabolism
GO:0006950	Response to stress
GO:0006350	Transcription

*Molecular Function *	
GO:0008233	Peptidase activity
GO:0005198	Structural molecule activity

AMX: amoxicillin; CTC: chlortetracycline; ENR: enrofloxacin.

**Table 3:** Differential *P. multocida* protein expression in response to a quarter-MIC AMX, CTC, and ENR in different GO categories.

Accession	GO Slims	∑Xcorr			
		Pm70	AMX	CTC	ENR
*Glycolysis/Gluconeogenesis *					
15603407	Phosphoenolpyruvate carboxykinase, PckA	264.2	ns	120	112.9
15603725	Phosphoglycerate kinase, Pgk	218.1	ns	170.2	161
15603726	Fructose-bisphosphate aldolase, FbaA	504.9	566.6	450.1	436.8
15602789	Glyceraldehyde-3-phosphate dehydrogenase, GapA	253.6	ns	125.5	70.1
15603371	Phosphoglyceromutase, GpmA	98.3	124.9	130.3	ns
15602518	Pyruvate kinase, PykA	28.6	51.4	54.9	ns
15602688	Fumarate hydratase, FumC	42.2	80.5	19.1	ns
15602146	SucD	bdt	7.8	ns	ns

*Response to stress *					
15602972	Chaperonin GroEL	148.6	246.8	ns	177.1
15602971	Co-chaperonin GroES	23	42.7	56.3	56.6
15602601	DnaK	97.6	132.7	ns	72.6
15602303	FtsH	21.9	7.9	4.7	3.9

*Peptidoglycon biosynthesis and cell envelope permeability *					
15603596	D-fructose-6-phosphate amidotransferase, GlmS	bdt	31.3	12.4	6.8
15603671	GlmU	8.5	20.2	ns	ns
15602548	NagC	14.4	ns	5.3	5
15602651	Hypothetical protein PM0786, OmpA	262.8	ns	172.5	76
15602419	Lpp	7.2	ns	18.7	ns

*Purine metabolism*					
15602149	Adenylate kinase, Adk	2.8	22.3	42.2	11.3
15602803	Adenylosuccinate synthetase, PurA	2.4	ns	11.2	19.2
15602109	Ribose-phosphate pyrophosphokinase, PrsA	bdt	9.2	8.4	ns

*RNA metabolism*					
15603255	DNA-directed RNA polymerase alpha subunit, RpoA	32.8	76.1	51.9	ns
15603602	DNA-directed RNA polymerase beta subunit, RpoB	17	34.5	ns	ns
15603601	DNA-directed RNA polymerase beta~ subunit, RpoC	56.2	85.2	ns	ns
15602786	DNA-directed RNA polymerase omega subunit, RpoZ	bdt	11	ns	9.6
15602119	Lrp	8.7	ns	25.1	ns
15603209	DeoR	bdt	10.6	ns	ns
15603785	Transcription termination factor Rho	42.4	15.9	20.5	21.3

*Xenobiotic metabolism*					
15601866	SodA	153.5	ns	66	85.4
15601897	HktE/KatE	26.1	ns	3	6.1

*Lipopolysaccharide biosynthesis*					
15603862	Lipid-A-disaccharide synthase, LpxB	bdt	12.1	ns	ns
15602423	2-dehydro-3-deoxyphosphooctonate aldolase, KdsA	9.2	23.2	ns	ns
15602749	RfaE	bdt	12.9	ns	ns
15603154	GalU	3.7	18.7	ns	ns

*Cell division*					
15603384	FtsY	bdt	8.2	ns	ns
15602012	Cell division protein FtsZ	11.5	22.1	ns	ns

*Protein translation*					
15603222	Elongation factor Tu	875.7	ns	936	ns
15602977	DeaD	4.7	ns	13.3	ns
15602168	Methionyl-tRNA synthetase	2.4	ns	11.5	ns

*DNA repair *					
15603682	Recombinase A, RecA	bdt	ns	ns	19.5

AMX: amoxicillin; CTC: chlortetracycline; ENR: enrofloxacin. Pm70: *P. multocida* cultured without sub-MICs. bdt: below detectable threshold. ns: differential expression not significant compared to Pm70.

## Abbreviations

MIC: Minimum inhibitory concentration
CTC: Chlortetracycline
AMX: Amoxicillin
ENR: Enrofloxacin
BHI: Brain heart infusion
GO: Gene ontology
NCBI: National Center for Biotechnology Institute
BRD: Bovine respiratory disease
BLAST: Basic local alignment search tool
ACN: Acetonitrile
2D: Two dimensional
LC: Liquid chromatography
ESI: Electrospray ionization
MS: Mass spectrometry
MS^2^: Tandem MS
RP: Reverse phase
RecA: Recombinase A
