# Supplementary material for: Effects of Subminimum Inhibitory Concentrations of Antibiotics on the Pasteurella multocida Proteome: A Systems Approach
Source: Comp Funct Genomics. 2008 Apr 22;2008:254836. doi: 10.1155/2008/254836 (PMC2367384; doi:10.1155/2008/254836)
Supplement: Supplementary file 3 [file 254836.f3.ppt]

## Slide 1
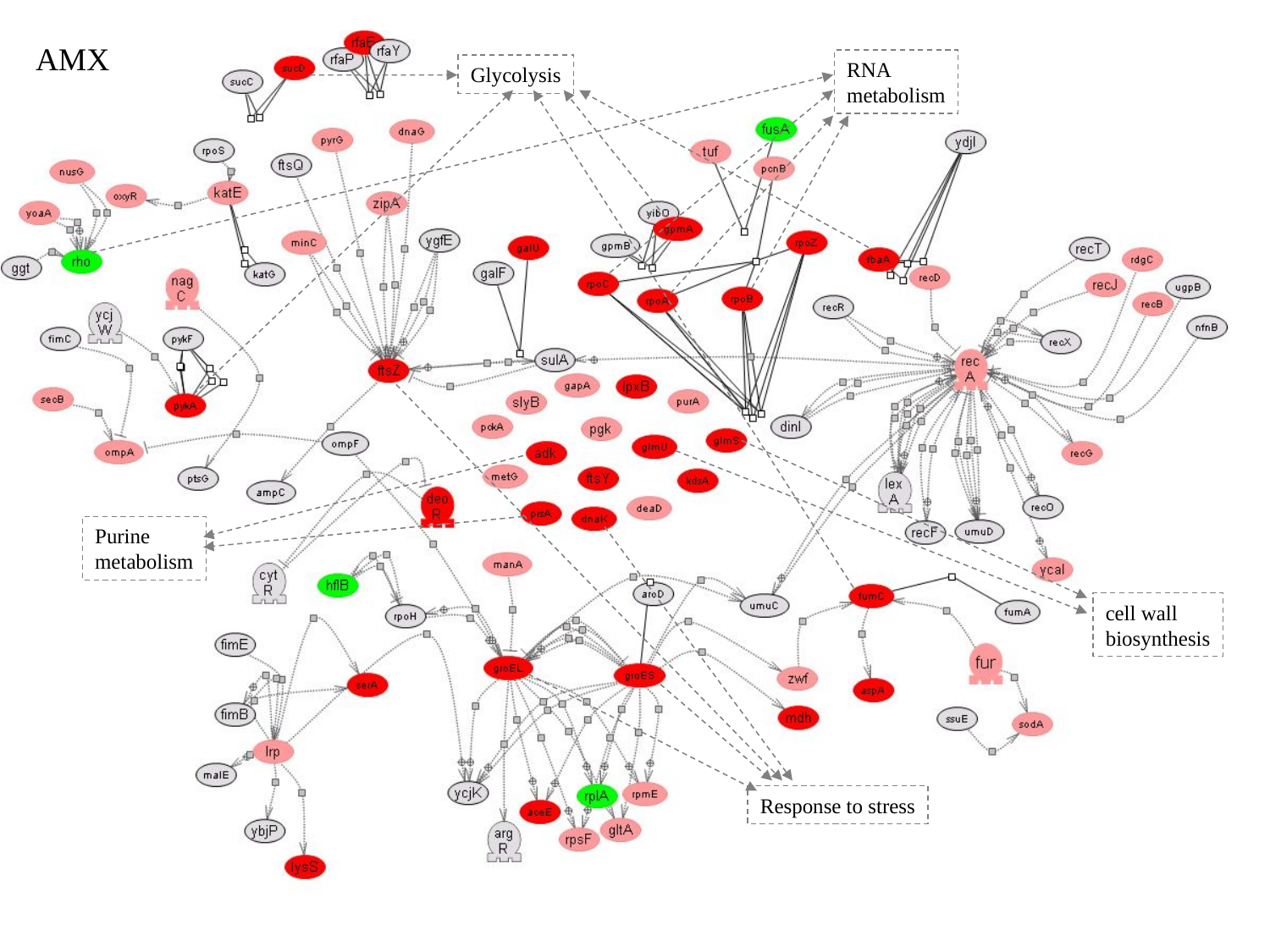

AMX
RNA
metabolism
Glycolysis
Purine
metabolism
cell wall
biosynthesis
Response to stress

## Slide 2
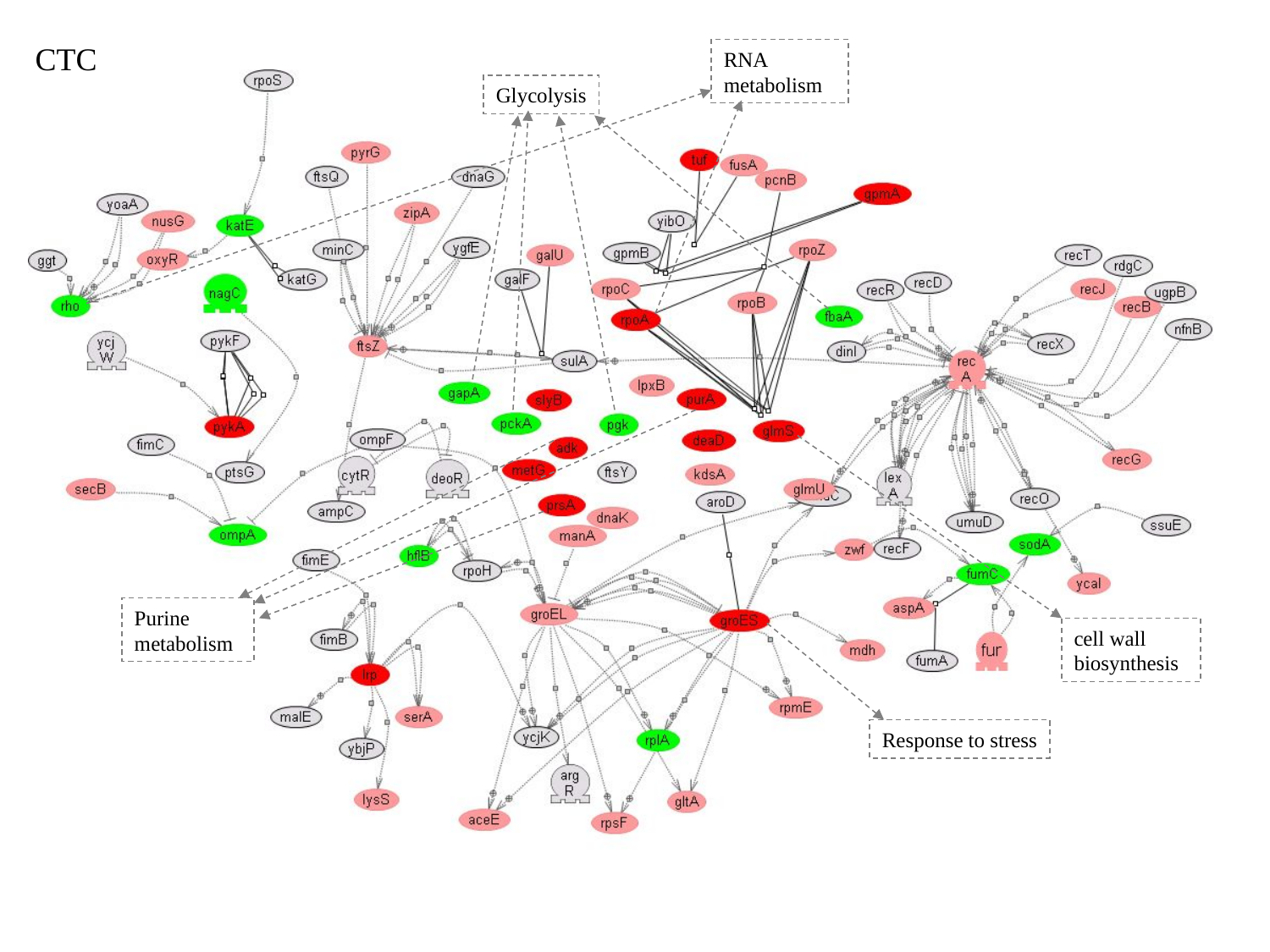

CTC
RNA
metabolism
Glycolysis
Purine
metabolism
cell wall
biosynthesis
Response to stress

## Slide 3
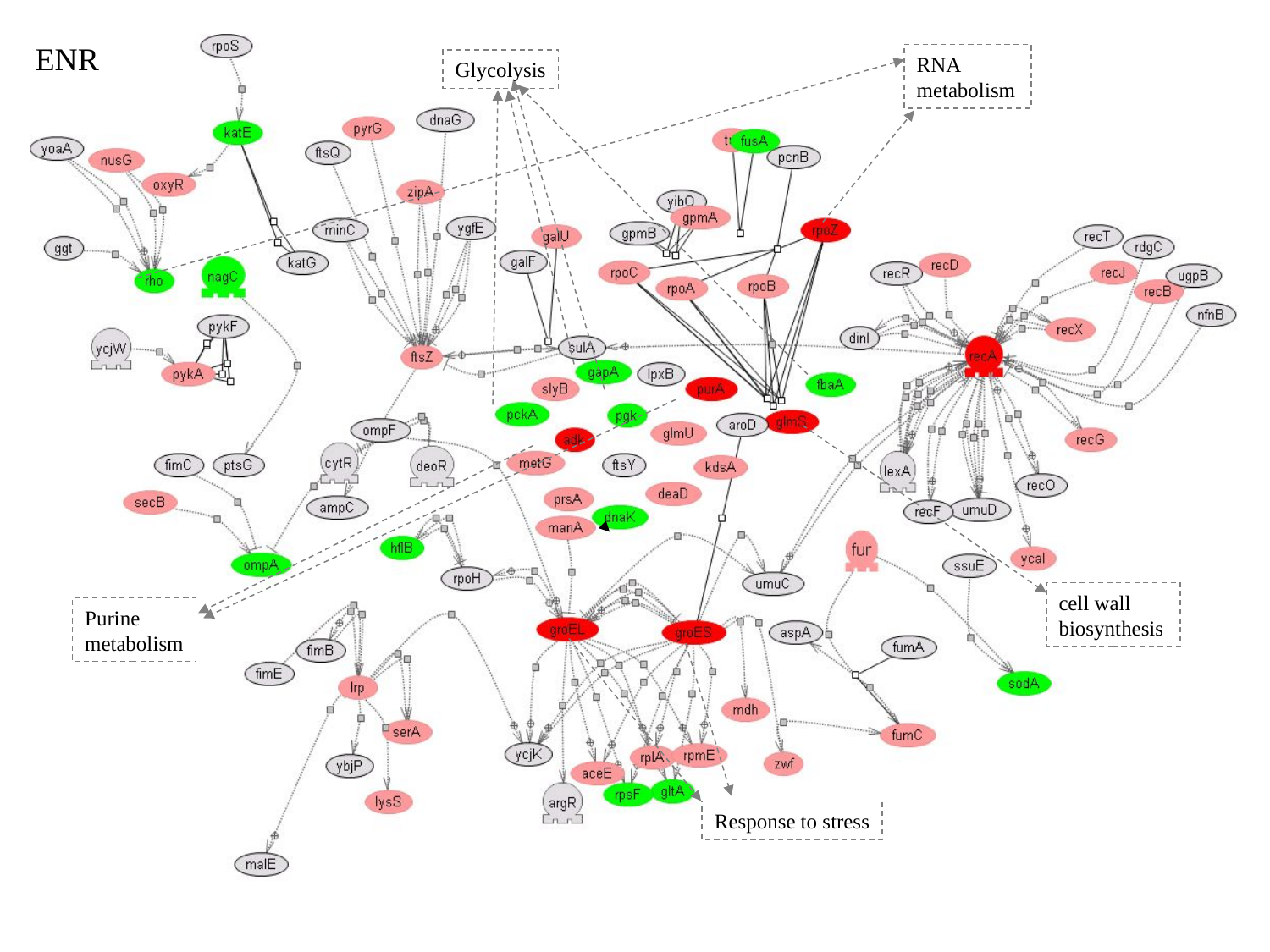

ENR
RNA
metabolism
Glycolysis
cell wall
biosynthesis
Purine
metabolism
Response to stress
